# A Comparison Between Clinical Guidelines and Real-World Treatment Data in Examining the Use of Session Summaries: Retrospective Study

**DOI:** 10.2196/39846

**Published:** 2022-08-16

**Authors:** Shiri Sadeh-Sharvit, Simon A Rego, Samuel Jefroykin, Gal Peretz, Tomer Kupershmidt

**Affiliations:** 1 Center for m2Health Palo Alto University Palo Alto, CA United States; 2 Eleos Health Waltham, MA United States; 3 Montefiore Medical Center Albert Einstein College of Medicine Bronx, NY United States

**Keywords:** Empirically based practices, natural language processing, psychotherapy, behavioral therapy, adherence, treatment fidelity, clinical training, real-world data, real-world study

## Abstract

**Background:**

Although behavioral interventions have been found to be efficacious and effective in randomized clinical trials for most mental illnesses, the quality and efficacy of mental health care delivery remains inadequate in real-world settings, partly owing to suboptimal treatment fidelity. This “therapist drift” is an ongoing issue that ultimately reduces the effectiveness of treatments; however, until recently, there have been limited opportunities to assess adherence beyond large randomized controlled trials.

**Objective:**

This study explored therapists’ use of a standard component that is pertinent across most behavioral treatments—prompting clients to summarize their treatment session as a means for consolidating and augmenting their understanding of the session and the treatment plan.

**Methods:**

The data set for this study comprised 17,607 behavioral treatment sessions administered by 322 therapists to 3519 patients in 37 behavioral health care programs across the United States. Sessions were captured by a therapy-specific artificial intelligence (AI) platform, and an automatic speech recognition system transcribed the treatment meeting and separated the data to the therapist and client utterances. A search for possible session summary prompts was then conducted, with 2 psychologists validating the text that emerged.

**Results:**

We found that despite clinical recommendations, only 54 (0.30%) sessions included a summary. Exploratory analyses indicated that session summaries mostly addressed relationships (n=27), work (n=20), change (n=6), and alcohol (n=5). Sessions with meeting summaries were also characterized by greater therapist interventions and included greater use of validation, complex reflections, and proactive problem-solving techniques.

**Conclusions:**

To the best of our knowledge, this is the first study to assess a large, diverse data set of real-world treatment practices. Our findings provide evidence that fidelity with the core components of empirically designed psychological interventions is a challenge in real-world settings. The results of this study can inform the development of machine learning and AI algorithms and offer nuanced, timely feedback to providers, thereby improving the delivery of evidence-based practices and quality of mental health care services and facilitating better clinical outcomes in real-world settings.

## Introduction

### Background

Mental health is a major global health concern. Mental illnesses will affect close to half of the world’s population at some point in their lives [1]. In addition to the personal toll of these illnesses, they also cost the global economy US $1 trillion per year in lost productivity alone [2]. Cognitive behavioral interventions have a robust evidence base for their efficacy and effectiveness in treating a variety of mental health issues. However, an ongoing challenge in the field has been how to “bridge the gap” between the laboratory or classroom and the clinic, with the data suggesting that the implementation of these methods in real-world settings is often much more challenging. One hypothesis for this is that therapists in real world settings may not be as adherent to the protocols on which these treatments have been tested and the evidence base established.

Cognitive behavioral treatments are designed to help individuals identify and alter maladaptive cognitions, emotions, interpersonal relationships, and problematic behaviors to reduce symptoms and lead more productive and satisfying lives. At this point, numerous versions of behavioral interventions have been manualized and studied in comparative trials with robust effects versus waitlist and placebo conditions, as well as medications and other forms of psychotherapy and disseminated worldwide. The need for focused behavioral treatments has become stronger following the COVID-19 pandemic, given the global increase in mental health disorders [2,3] and the resulting demand for mental health services, coupled with a dramatic shortage of clinicians [4]. Given this need-service gap, it is imperative that behavioral treatments be delivered in accordance with the empirically based guidelines based on which they were established in order to maximize the potential of replicating the outcomes of the clinical trials with individuals “in the real world” with mental health concerns.

### Therapist Adherence to Treatments as Designed

“Therapist drift,” or the tendency of clinicians to adhere only partially to established empirically supported practices, has been documented in the literature with respect to many treatment models [5,6]. Therapists’ attitudes toward evidence-based practices (EBPs), their licensing status, and organization characteristics combinatorically affect their use of recommended strategies [7,8]. Further, clinical trainings or specialty workshops for EBPs are often not enough to facilitate change of practice and improved client outcomes unless some ongoing follow-up sessions are offered for supervision [9].

Complicating matters further, therapy is often like a “black box” in which little is done to monitor or encourage clinicians to adhere to core active ingredients of empirically supported treatments. As such, it has been proposed that clinical research should utilize observations that are less subject to bias [10,11]. In addition, data from real-world treatments indicate that few empirically based therapeutic techniques are in effect, even when clinicians report that they offered evidence-supported behavioral treatments [12].

### Session Summary: An Example of Therapist Adherence

Empirically supported protocols are often built on a basic set of theoretical ideas. Although certain aspects may differ amongst protocols, certain essential concepts remain common to them all [13]. Dryden [14] argues that it is essential for the client to leave the session with what they view as important takeaways. As the session comes to a close, it is best practice for the therapist to invite the client to summarize the session rather than the therapist summarizing what transpired for the client during the session [14]. Indeed, one of the key elements in almost every manualized treatment is encouraging the client to compose a *summary* at the end of each therapy session [15-19]. This form of feedback is described as a means of helping clients review their own understanding of the session and the rationale for the interventions provided [20]. Further, a session summary provides clients an opportunity for feedback, which empowers clients to conceptualize their needs, assess their progress toward their goals, and make their own decisions. It also allows the therapist to ensure that key components of the sessions have been understood and highlighted. Therefore, a session summary could be viewed as a common, transdiagnostic evidence-based component that is one of the key ingredients related to the effectiveness of the session.

The importance of session summary has not been overlooked by researchers, and reports on treatment studies often mention this strategy [21,22]. Perlich and Meinel [23] even developed a tool for collaborative session summary, in which the client and the therapist review the session and their takeaways. However, when asked about their own adaptations of EBPs, 32% of therapists reported removing components of the intervention, with the session summary being the most frequently omitted part from the therapy process [24]. Similarly, one of the most common challenges of community mental health therapists learning cognitive behavioral therapy (CBT) was their limited attempts to solicit the client’s feedback [20], and in text-based CBT, very few therapists summarized the session [25].

While the session summary is just one example of possible therapist drift [6], given its importance of reinforcing the therapeutic process and strengthening the learnings obtained during treatment, and that it is a technique that can be delivered quite briefly and has a high face validity across interventions, it may serve as a proxy for how much the clinician is adhering to the full range of tasks that are essential for evidence-based therapies (EBTs) to be effective. Therefore, this study explored how common are session summaries in real-world behavioral treatments.

## Methods

### Settings and Interventions

This study is based on the retrospective analysis of fully anonymized data from behavioral health treatments provided in 37 behavioral health care programs across the United States. All client participants received either individual, group, or couples therapy in either an outpatient or intensive outpatient program. Clients sought treatment for a range of mental health concerns, and therapists were free to provide the intervention they believed was most suitable for the client’s presenting problem and characteristics. Therapists were either psychologists, social workers, or licensed counselors. The sample comprised 17,607 treatment sessions administered by 322 therapists to 3519 clients.

### The Eleos Health Platform

All sessions were processed via an artificial intelligence (AI) therapy-specific platform (Eleos Health). This platform captures the treatment conversations, provides a verbatim session transcript, and summarizes intervention insights to inform treatment-planning and clinical decision-making [26]. The platform collects key metrics from treatment sessions and integrates them with standardized evidence-based self-report measures, leveraging insights developed through machine learning (ML) and natural language processing (NLP) analysis of large treatment data sets [27]. Eleos also uses AI methods to increase adherence with clinical standards and drive operational efficiency.

### Ethical Considerations

The platform is Health Insurance Portability and Accountability Act–compliant, and all participants consented to have their sessions processed through it. This study was approved by an external institutional research board, Sterling IRB (9545).

### Data Processing

To fully capture and make sense of the speech data, we developed an algorithm to identify the specific interventions carried out in behavioral treatment sessions and consequently determine whether a session summary was recorded. First, the sessions were transcribed using an automatic speech recognition system (ASR) as well as a domain-based text-cleaning algorithm. Second, since the session transcripts are unstructured data, we developed a treatment-oriented, speaker diarization ML model in accordance with their utterances in the treatment session. A team of trained graduate-level clinicians tagged over 2500 therapy conversations and labeled the speakers as either “patient” or “therapist.” These data were consequently used in a model that analyzed the full transcribed session and assigned a speaker label for each participant. Third, we applied the term frequency–inverse document frequency, a commonly used feature generation method, to identify if the speaker is either the therapist or a client. As a classification algorithm, we used a logistic regression model with a binary cross-entropy loss and trained the model using stochastic gradient descent. On a session level, our in-house solution demonstrated 98% accuracy in differentiating between speakers in therapy sessions.

### Data Analysis

**Figure 1:**
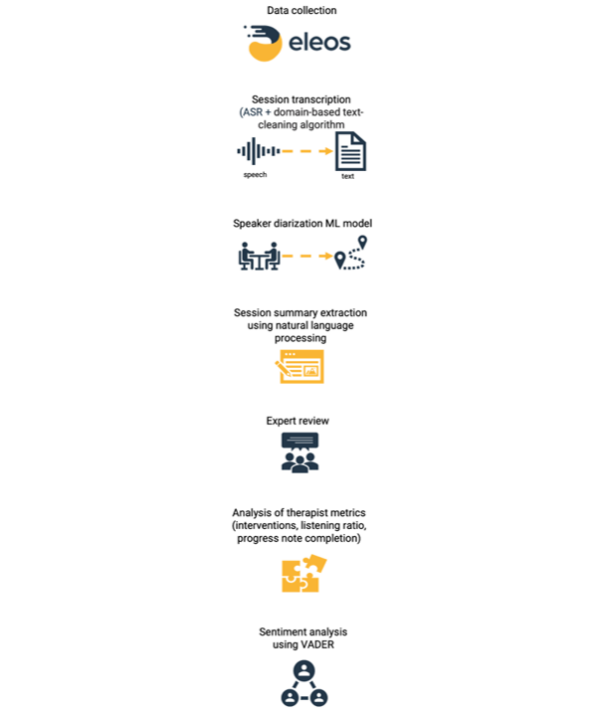
Overview of the classification and data analysis process for real-world session summaries. ASR: automatic speech recognition; ML: machine learning; VADER: Valence Aware Dictionary and sEntiment Reasoner.

Eleos’s NLP-based engine extracted potential session summary from therapists’ utterances, identifying the frequencies of lexical terms, which were said during the latter 20% of the session, and retrieving phrases such as the following: “[I] just want to review what we talked about“; “So what did you learn here today?”; “If it was something that you were going to take away from today's session, what would it be?“; “What's your take home message from today?”; “Alright, let's do our summary for the day“; “'kind of [your] two main takeaways”; and so on. For quality assurance, 2 psychologists (SSS and TK) reviewed 682 sessions that included language associated with a session summary and indicated whether the algorithmically identified text did in fact reflect a meeting review prompt. Figure 1 outlines the data analysis approach used in this study. Finally, we compared sessions with and those without a summary on the following variables: most commonly discussed topics, therapist-to-client listening ratio, the most commonly used intervention techniques, and content of the progress note that the therapist had generated for this session in the program’s electronic health record (EHR). Further, to assign the sentiment expressed during these specific sessions, we applied Valence Aware Dictionary and sEntiment Reasoner (VADER) [28] on the therapist’s and patient’s texts, independently. VADER is a lexicon and rule-based sentiment analysis tool that uses a sentiment lexicon and a list of lexical features (eg, words) that are labeled in accordance with their semantic orientation as either positive or negative [29]. VADER not only classifies the data to either positive or negative, but also provides a score to indicate the strength of the sentiment detected [28].

## Results

### Provision of a Session Summary

Our analysis found that only 54 of the 17,607 (0.30%) behavioral treatment sessions included a session summary. Of these 54 treatment meetings, session summaries most commonly addressed interpersonal relationships with family and friends (n=27), issues related with work (n=20), the word “change” (n=6), and alcohol (n=5).

### Characteristics of Sessions Including Summaries

Data were further analyzed to review descriptive differences between therapy sessions with and those without a summary. Sessions that included a prompt to help the client summarize the meeting had a lower therapist listening ratio (33% vs 49%), indicating that therapists were less verbally active throughout the meetings that included feedback. The number of therapeutic interventions detected was greater in sessions that included a session summary; on average, therapists used 17% more interventions in meetings with a summary. Sessions with meeting summaries also included greater therapist use of validation, complex reflections, and proactive problem-solving techniques. Moreover, therapists who prompted their client to summarize their session were 83.3% more likely to assign treatment homework and report it on their EHR progress note. Therapists who encouraged a summary also had a 12% greater likelihood of completing their progress note within 48 hours of the session’s date: 69% of those who had asked for a summary also completed their progress note within this time frame, compared to 61% of therapists who did prompt a summary. Table 1 provides an overview of the differences between sessions including and not including summaries.

Applying the VADER algorithm in sessions that included summaries revealed that the clients expressed, on average, slightly more positive and negative emotions (0.6% and 4%, respectively) than those with no summary. However, the therapists tended to express less emotion in the sessions including a summary, expressing 9% less positive statements and 7% less negative statements. Table 2 provides an overview of the differences between sessions including and those not including summaries around statements’ sentiments.

**Table 1:** Differences in therapists’ behaviors between sessions including and those not including a meeting summary.

Sessions	Listening ratio, %	Progress note completion rate, %	Type of interventions detected
Without a summary	33	61	1.04
With a summary	49	69	1.26

**Table 2:** Sentiment differences between sessions including and those not including a meeting summary.

Sessions	Patients’ positive statements, %	Patients’ negative statements, %	Therapists’ positive statements, %	Therapists’ negative statements, %
Without a summary	7.70	4.50	8.90	4.20
With a summary	7.40	4.50	8.20	3.90

## Discussion

### Principal Findings

While the evidence base is strong and robust for behavioral interventions, their efficacy is tied to maintaining a structure and including certain key components in each session. Therapist drift from the key active ingredients of validated treatment protocols could compromise the efficacy and effectiveness of the treatments, thus limiting the impact of treatment on the individual [30]. This study examined the practice guidelines versus practice in real-world behavioral health care settings as they pertain to a key component found in most behavioral interventions: encouraging clients to review and summarize their treatment session [20]. Session summaries are important because they allow the client an opportunity to reflect back on the treatment meeting, their developing understanding of the maladaptive processes underlying their symptoms, as well as some effective coping strategies they could employ. They also allow the therapist to ensure that key components of the sessions have been understood and highlighted. In controlled and case-series studies reported in the literature, the technique of requesting feedback is stated explicitly [31]. This study found that very few therapists provide feedback to their clients in the form of a session summary. Our findings suggest that providers who encouraged their clients to reflect on their treatment demonstrated a more active therapy style—their sessions were characterized with more back-and-forth exchanges between the therapist and the client, they provided more interventions during the meeting, and they even tended to complete their progress note faster. These results suggest that therapist adherence to at least one of the key components of most empirically supported behavioral treatments was absent in most of the real-world sessions we reviewed.

### Comparison With Prior Work

The findings of this study indicate that in contrast to guidance in treatment protocols, therapists delivering behavioral treatments in real-world settings rarely encourage their clients to reflect on the session during their meeting. To the best of our knowledge, this study is the first to evaluate a large and diverse data set of actual therapy sessions. These findings extend the results of previous studies that have exclusively relied on practitioner self-report and provide insight on how therapists practice in real-world treatment settings [32]. Therapists may overestimate their adherence to practice guidelines, as 32% reported not providing all parts of treatments [32], while this study suggests that adherence rates are much smaller. Session summary, or feedback, can be perceived as a method for prompting clients to form implementation intentions, thereby likely facilitating greater treatment impact; however, prior research has found that therapists do not often explicitly discuss with their clients to plan actions as a result of the treatment session [33]. Further, higher-caseload therapists reported feeling that learning about new EBTs would be time-consuming, which consequently could serve as a barrier to implementing these techniques [30]. In light of this research, it may not be surprising that therapists do not adhere to EBT recommendations despite realizing their potential benefits to service users. Of note, it has been proposed in the literature that treatment protocols are difficult to administer in the field as originally designed in controlled studies, and that “flexibility within fidelity” should be practiced in order to maximize the effects of these programs [13]. Hence, a systematic understanding of the context affecting variations from prescribed practice and omissions of specific techniques is warranted.

### Limitations

This study utilized data from 17,607 sessions taking place in behavioral health clinics across the United States. The data are likely more representative of the therapist behaviors occurring in real-world settings than are the findings of controlled studies. Nonetheless, this study has limitations. The anonymized database did not include demographic and clinical information of the clients and therapists, which could have enriched our analysis. Future studies should also collect explicit data on the treatment that was provided and how it maps on to the client’s treatment plan. Further, the low number of sessions with summary statements limited our ability to utilize the sentiment and content analyses. Additionally, the analysis did not include outcome data such as symptom reduction or client satisfaction, which are important to assess in the context of the treatment process. From a theoretical and practical standpoint, interviewing therapists about their considerations of using strategies will help better define underlying processes affecting behavioral treatment implementation.

### Conclusions

Given the importance of following treatment protocols as initially intended, there is much potential in automating timely feedback for therapists. This study is the first to our knowledge that provides real-time, observational data on clinical practice in real-world settings. As such, it provides a new perspective to how clinicians provide therapy that can enrich that data captured by therapist self-reports. Empirically supported ML and AI algorithms can offer clinicians, trainers, supervisors, and stakeholders nuanced observations on treatment adherence, thereby improving the quality of implementation, dissemination, and ultimately, effectiveness of mental health treatments.

## Abbreviations

AI: artificial intelligence
CBT: cognitive behavioral therapy
EBP: evidence-based practice
EBT: evidence-based therapy
EHR: electronic health record
ML: machine learning
NLP: natural language processing
VADER: Valence Aware Dictionary and sEntiment Reasoner

## Data Sharing Statement

The data that support the findings of this study are not publicly available owing to privacy and ethical restrictions.
